# Response to the letter to the editor

**DOI:** 10.1007/s10006-026-01589-7

**Published:** 2026-07-16

**Authors:** Alina Marie Schmitz, Nils Lucca Kern, Homam Ward, Marius Sauerbrey, Friedrich Mrosk, Oliver Wagendorf, Susanne Nahles, Carsten Rendenbach, Norbert Neckel, Max Heiland, Steffen Koerdt

**Affiliations:** Department of Oral and Maxillofacial Surgery, Charité – Universitätsmedizin Berlin, corporate member of Freie Universität Berlin and Humboldt-Universität zu Berlin, Augustenburger Platz 1, Berlin, 13353 Germany

Dear Prof. Schliephake,

We would like to thank Mr. Gholami and his colleagues for their interest in our study and for their thoughtful comments.

We agree that culture-based pathogen detection does not reflect the full microbial diversity of odontogenic infections. As we discussed in our manuscript, the complexity of culture-based methods should be interpreted as a pragmatic measure derived from routine clinical microbiology, rather than as a comprehensive assessment of the oral microbiome. Our study aimed to investigate whether the number of cultured pathogens is associated with clinically relevant microbiological outcomes, rather than characterizing the complete microbial ecosystem.

We also agree that immunosuppression is a heterogeneous group of conditions. This was acknowledged as a limitation in the discussion section. Due to the retrospective design of the study and the small number of patients within individual immunosuppressed subgroups, a broader definition was chosen. Future studies with larger cohorts may help to clarify whether specific forms of immunosuppression are associated with distinct microbiological patterns.

The authors also highlight the limitations of complete-case analysis. We agree that loss of patients due to missing covariate data is an important consideration. However, we would note that the main findings remained consistent across both the fully adjusted and sensitivity analyses. Although the magnitude of the effect estimates differed, a higher level of microbial complexity was strongly associated with polymicrobial infection, *Enterobacterales* detection and antimicrobial resistance in all models.

Reverse causation and residual confounding are still possible explanations that should be addressed in future prospective studies.

Overall, we appreciate the comments by Mr. Gholami et al. We agree with their suggestion that future studies using molecular microbiological methods and prospective study designs are important for further investigating the relationship between microbial community structure, antimicrobial resistance and host-related factors in odontogenic infections.

Sincerely,

Alina M. Schmitz.

On behalf of all authors.

## Data Availability

No datasets were generated or analysed during the current study.

